# 

**Published:** 1890-04

**Authors:** 


### CONTENTS—APRIL, 1890.—VOL. 5, NO. 10.

ORIGINAL COMMUNICATIONS— Page

Puerperal Convulsions. By S. H. Stout, A. M., M. D., LL. D., Cisco . . . . . . . . . . . . . . . . . . . . 365

Peri-Typhlitis, with Abscess of Appendix Vermiformis; Operation and Recovery. By W. A. Ellison, M. D., Manchaca . . . . . . . . . . . . . . . . . . . . . . 376

Some Amusing Theories of By-gone Days. By E. Wildeman, M. D . . . . . . . . . . . . . . . . . . . . . 381

Successful Case of Laparotomy. By R. E. Moss, M. D . 383

CORRESPONDENCE—

Screw Morms in the Mouth. Letter from Dr. Cross . . 384

SOCIETY NOTES—

Minutes of the Austin District Medical Society. [Read it, as a point in ethics is there decided.] . . . . . . . . . 385

Revision of the U. S. Pharmacopœia—Seventh Decennial Convention . . . . . . . . . . . . . . . . . . . . . 387

Arrangements for the Meeting at Fort Worth, April 22, 1890 . . . . . . . . . . . . . . . . . . . . . 388 and 397

EDITORIAL—

The Twenty-Second Annual Convention of the Texas State Medical Association . . . . . . . . . . . . . . 389

Some Reasons why Physicians should attend a Southern Polyclinic . . . . . . . . . . . . . . . . . . . . . 392

BOOK NOTICES—

Annual—Universal Medical—Science—Foods for the Fat —A Laboratory Guide . . . . . . . . . . . . . . . 393

MEDICAL NEWS AND MISCELLANY—

Deaths—Marriages—Removals, etc . . . . . . . . . . . 395

Circular to Confederate Veterans of the Medical Corps . . 399

PUBLISHER'S NOTES—

Of Interest to Physicians and Others . . . . . . . . . . 401

